# Comparative analysis of anthropometric indices of obesity as correlates and potential predictors of risk for hypertension and prehypertension in a population in Nigeria

**DOI:** 10.5830/CVJA-2016-061

**Published:** 2017

**Authors:** Chimaobi James Ononamadu, Godwin Okwudiri Ihegboro, Chinwe Nonyelum Ezekwesili, Onyemaechi Faith Onyeukwu, Uchenna Francis Umeoguaju,, Obiajulu Christian Ezeigwe

**Affiliations:** Department of Biochemistry and Forensic Science, Nigerian Police Academy, Wudil, Kano State, Nigeria; Department of Biochemistry and Forensic Science, Nigerian Police Academy, Wudil, Kano State, Nigeria; Department of Applied Biochemistry, Nnamdi Azikiwe University, Awka Anambra State, Nigeria; Department of Applied Biochemistry, Nnamdi Azikiwe University, Awka Anambra State, Nigeria; Department of Applied Biochemistry, Nnamdi Azikiwe University, Awka Anambra State, Nigeria; Department of Applied Biochemistry, Nnamdi Azikiwe University, Awka Anambra State, Nigeria

**Keywords:** hypertension,, prehypertension,, obesity,, anthropometric index,, ROC curve

## Abstract

**Background:**

Obesity is a well-established independent risk factor for hypertension and other cardiometabolic disorders. However, the best anthropometric index of obesity that predicts or associates strongly with hypertension and related conditions remains controversial and inconclusive.

**Objective:**

This study compared the performance of eight anthropometric indices of obesity: body mass index (BMI), ponderal index (PI), waist circumference (WC), hip circumference (HC), waist–hip ratio (WHR), waist–height ratio (WHtR), body adiposity index (BAI) and conicity index (CI) as correlates and potential predictors of risk of hypertension and prehypertension in a Nigerian population, and also the possible effect of combining two or more indices in that regard.

**Methods:**

This church-based, cross-sectional study was conducted in Anambra state, south-eastern Nigeria from 2012 to 2013. A total of 912 persons (436 male and 476 female) drawn randomly from three major cities (Awka, Onitsha and Nnewi) in the state participated in the study. Information on demography, medical history and lifestyle were obtained using a well-structured and validated questionnaire. The systolic/diastolic blood pressure and anthropometric measurements were taken by well-trained personnel. The resulting data were analysed using descriptive statistics, logistic regression, Poisson regression and receiver operating characteristic curve analysis.

**Results:**

The mean values of all the anthropometric indices studied increased from normotension, through prehypertension to hypertension in both genders. BMI, WC, HC and CI were significantly higher (p < 0.05) in females than males. All the anthropometric indices studied were significantly (p < 0.001 except for CI) correlated with systolic and diastolic blood pressure. BMI, WHtR, WC and PI (with higher correlation coefficients for blood pressure) showed the best potential potential to predict hypertension and prehypertension in the study: BMI (cut-off = 24.49, AUC = 0.698; cut-off = 23.62, AUC = 0.659), WHtR (cut-off = 0.55, AUC = 0.682; cut-off = 0.5, AUC = 0.636), WC (cut-off = 91.44, AUC = 0.692; cut-off = 82.55, AUC = 0.645), PI (cut-off = 14.45, AUC = 0.670; cut-off = 13.69, AUC = 0.639), in males; and BMI (cut-off = 24.44, AUC = 0.622; cut-off = 28.01, AUC = 0.609), WHtR (cut-off = 0.51, AUC = 0.624; cut-off = 0.6, AUC = 0.572), WC (cut-off = 96.62, AUC = 0.616; cut-off = 96.52, AUC = 0.584), PI (cut-off = 16.38, AUC = 0.619; cut-off = 17.65, AUC = 0.599), in females for hypertension and prehypertension, respectively. In predicting hypertension risk, WC and WHtR did not significantly improve the performance of BMI in the models when included using our decision rule. Overall, CI had a very poor discriminatory power for both conditions in this study.

**Conclusion:**

BMI, WHtR, WC and PI emerged the best predictors of hypertension risk, and BMI, WC and PI of prehypertension risk in this study. The combination of high-performing anthropometric indices in a model did not improve their performance. Therefore we recommend the simultaneous but independent use of BMI and either WC or WHtR for predicting hypertension, and BMI and WC for prehypertension risk, bearing in mind that both types of index (abdominal and general obesity) account for different forms of obesity.

## Background

The burden of the metabolic syndrome, which includes hypertension, is rising to epidemic proportions in Africa at present. According to the World Health Organisation (WHO) health report in 2001, cardiovascular diseases alone accounted for 9.2% of the total deaths in Africa, killing more people than even malaria.^1^

Hypertension and prehypertension are considered risk factors for cardiovascular and coronary heart disease. The prevalence and diagnosis of hypertension in children and adolescents appears to have increased in recent times.^1^ Prehypertension is considered a new category of hypertension and a major risk factor for developing clinical hypertension relative to those with normotension. It is characterised by blood pressure levels slightly higher than normal. Research in this area has suggested that exploration and modification of risk factors could help ameliorate this trend.^1^

Obesity is a disorder characterised by extensive fat accumulation, and the body fat is distributed in such a way that health and wellbeing are affected.^2^ The condition is an established risk factor for hypertension, hypercholesterolaemia, insulin resistance and diabetes.^3^,^4^ The close association of obesity with blood pressure has long been recognised in both genders and even in diverse racial/ethnic groups.^5^

Anthropometry is the most basic method for assessing body composition. It describes body mass, size, shape and level of fatness.^6^ It is an easy, economical and effective method that is used in the initial screening of obesity, hypertension and other metabolic disorders.^7^

Research efforts have developed many anthropometric indices to specifically describe obesity and fat distribution in humans. These include body mass index (BMI), ponderal index (PI), waist circumference (WC), hip circumference (HC), waist–hip ratio (WHR), waist–height ratio (WHtR), body adiposity index (BAI) and conicity index (CI).

BMI is promulgated by the WHO as the most useful epidemiological measure of obesity, but its usefulness suffers from its inability to account for body fat distribution.^8^ BMI and PI are widely used to describe total or general obesity, while WC, WHR, WHtR and CI describe more visceral fat; abdominal or centralised obesity.^9^,^10^ Anthropometric indicators of abdominal obesity can provide estimates of the visceral adipose tissue, which in turn is associated with metabolic changes, hyperinsulinaemia, glucose intolerance, hypertriglyceridaemia and hypertension.^11^

The direct association between hypertension and anthropometric indices of obesity have been studied in many countries and ethnic groups,^7^ but results from different studies show that the best anthropometric index in predicting hypertension and other components of the metabolic syndrome remains inconclusive and controversial.^7^,^12^,^13^ Some studies reported that the best single indicator of the risk of hypertension in Japanese and Cuban populations was BMI.^3^,^14^ Other studies suggested WC was a better predictor in Greek,^15^ Taiwanese (women) and some Japanese men.^7^

WHtR has also been suggested as the best predictor of hypertension for elderly men in Barbados,^14^ Taiwanese men,^16^ and Korean men,^17^ whereas other studies demonstrated that WHR was the best predictor for Argentinian men and women,18 and indigenous Australian men and women.19 Lee and Kim,^7^ and Fuchs et al.^13^ suggested that combination of two or more indices could improve the predictive power of an index. Different studies have therefore posited that the predictive power of an anthropometric index may be population-dependent and vary across ethnic groups, age and gender.^20^,^21^

In Nigeria, studies that assessed the performance of anthropometric indices in predicting risk of some metabolic disorders such as hypertension are lacking. Okereke et al.^22^ evaluated the anthropometric indices for the diagnosis of obesity in pregnant women in Nigeria, and Okafor et al.^23^ compared the performance of WC and WHR. However, no study has comprehensively assessed the performance of anthropometric indices of obesity in predicting hypertension and prehypertension.

Based on this premise, our study intended to compare the performance of eight anthropometric measures of obesity: BMI, PI, WC, HC, WHR, WHtR, BAI and CI as indicators of risk for hypertension and prehypertension, and the effect (on the performance) of combining two or more of the best-performing indices.

## Methods

The study was a cross-sectional, church-based survey carried out in three major cities in Anambra state. The inclusion criteria were: age range 17–79 years, being a resident in the study areas Awka, Nnewi and Onitsha cities of Anambra state, being selected by the random sampling procedure explained below, providing informed consent/willingness to participate, and complying with the instructions of the study, for example, avoidance of alcohol, coffee, drugs and exercise at least 30 minutes before examination.

Information on the prevalence of hypertension in the adult and paediatric population of Anambra state was lacking, but based on data in the literature, the prevalence in Nigeria ranges from 8–30%.^24^ Using the ‘stat calc’ function of Epi INFO (version 7) software, it was determined that a sample size of 900 was adequate to detect the prevalence of hypertension of 10–30% with 3% precision and 95% confidence.

A total of 1 000 participants (we lost 88 to follow up on the day of testing and administration of the questionnaire) were randomly selected from 30 primary sampling units. A stratified random sampling technique was employed. In brief, the three major cities, Awka, Nnewi and Onitsha were selected for this study. The cities were stratified by location (rural versus urban areas) to ensure good representation. Since these populations are predominantly Christian, the survey was made church based. The churches constituted the primary units from which individuals or participants were randomly sampled; 10 churches from each city.

Firstly, pre-visits to the three cities provided us with a list (dataset) of known churches in the communities within the cities. For each city, six and four churches were randomly selected from the urban and rural areas, respectively, using the ‘sample, count’ command of Stata statistical package. A total of 30 churches were selected from a total of 224 churches (Awka 71, Onitsha 90, Nnewi 63). The urban areas were more populated and therefore were sampled more. The selected churches were visited. A list of members who showed willingness to participate was made after explaining the objectives and nature of the study to the congregation, and 11 participants were randomly selected using the ‘sample, count’ command of Stata.

Information on demography and lifestyle was obtained using a well-structured and validated questionnaire. Anthropometric data, which included weight, height, and waist and hip circumferences were obtained by well-trained personnel. Weightwas measured to the nearest 0.5 kg using a weighing scale with the participant removing his/her footwear. Height was measured to the nearest 0.5 cm using a local stadiometer fixed to a wall. The waist circumference was measured at the level of the iliac crests,^25^ using a flexible tape and passing it along the umbilical level of the unclothed abdomen. The hip circumference was measured around the widest portion of the buttocks, with the tape parallel to the floor.

Blood pressure was taken from the non-dominant arm after 15 minutes of rest, using appropriate cuff size and Accoson brand of mercury sphygmomanometer. Systolic (SBP) and diastolic blood pressure (DBP) were the first and the fifth koroktoff sounds, respectively. Three consecutive measurements were made at an interval of five minutes after a 10-minute rest. The mean SBP and DBP determined from the second and third measurements were used for data analysis.

Anthropometric indices were calculated as follows: BMI = weight (kg)/height^2^ (m); WHR = WC (cm)/HC (cm); WHtR = WC (cm)/height (cm); PI = weight (kg)/height^3^ (cm); BAI = HC (cm)/height^1.5^ (m) – 18; CI = ^WC (m)^/_(0.109 × weight (kg)/height (cm))_

Hypertension was defined using the WHO/ISH criteria of SBP ≥ 140 mmHg and/or DBP ≥ 90 mmHg, or clinical diagnosis of hypertension, or prescription of any hypertensive drug. Prehypertension was defined as SBP ≥ 120 mmHg and/or DBP ≥ 80 mmHg.

This study was conducted with adherence to ethical standards. Ethical approval was obtained from the ethics committee of Nnamadi Azikiwe Uiversity, Awka, Nigeria. The objectives and nature of the study were duly explained to the participants prior to the day of the test and interview. Informed consent in written form or by thumb print was obtained from all participants or parents. Strict confidentiality was maintained in accordance with standard medical practices.

## Statistical analysis

Comparison of means between the two groups was done using the independent t-test. Poisson regression models were used to examine the association between anthropometric indices and hypertension/prehypertension prevalence. The receiver operating characteristic (ROC) curve analysis was used to compare the performance of the anthropometric indices as potential predictors of the disease. The ROC curve is an analytical approach to define the highest combination of sensitivity and specificity of a screening test. The approach has been widely used to determine a cut-off point for decision making (e.g. having a disease or not) in both public health and clinical settings.

Area under the curve (AUC) was used as a measure of predictive power. It is the most common measurement to quantify the performance of a screening test, and shows the ability of a test to correctly classify those with and without the disease. For example, an AUC of 0.75 indicates that 75% of the time, a randomly selected individual from the diseased group has a test value larger than that for a randomly selected individual from the non-diseased group. AUC values range from 0.5 (no prediction) to 1.0 (perfect prediction). AUC values are usually used as criteria to compare overall performance of different screening tests. In this study, AUCs for models were estimated using logistic regression models.

To determine if the inclusion of WC, WHtR or PI improved the prediction of hypertension using BMI, we estimated the change in gender-specific prevalence ratio (from Poisson regression models) and gender-specific AUC (from logistic regression models) between a model with BMI + WC, BMI + PI or BMI + WHtR to a model with BMI alone, as described by Tuan et al.^28^ A change in prevalence ratio or AUC of ≥ 10% was used as the criterion for a significant contribution of WC, PI, or WHtR to the prediction of hypertension using BMI. This criterion of ≥ 10% was adopted as it is commonly used to determine a notable confounding factor.^26^

Data analysis was conducted using Stata and MedCalc statistical packages. Model 1 was crude while model 2 was adjusted for factors, such as age, smoking, alcohol consumption and physical activity.

## Results

A total of 912 individuals aged 17 years and older from the three major cities participated and provided informed consent for the study; 32.89% of the respondents were from Awka, 33% from Onitsha and 34.10% from Nnewi. The overall crude prevalence of hypertension and prehypertension in the study population was 22.81 and 42.54%, respectively.

Tables 1 and 2 show the general characteristics of our study population. The mean values of all the anthropometric indices analysed were significantly higher in the women, with the exception of weight, height and WHR, when compared to the men. The mean values of all anthropometric indices studied also increased from normotensive participants, through prehypertensive subjects and peaked in the hypertensive participants in both male and female categories, with hypertension showing the highest mean values for all anthropometric indices studied.

**Table 1:** General characteristics of the study population

Variables	No hypertension, n (%)	Hypertension, n (%)
Age (years)		
≤ 20	92 (10.09)	7 (0.77)
21–25	279 (30.59)	29 (3.18)
26–40	245 (26.86)	90 (9.87)
≥ 41	88 (9.65)	82 (8.99)
City		
Awka	241 (26.43)	59 (6.47)
Onitsha	203 (22.26)	98 (10.75)
Nnewi	260 (28.51)	51 (5.59)
Gender		
Male	340 (37.28)	96 (10.53)
Female	364 (39.91)	112 (12.28)
Body mass index		
Underweight	13 (1.43)	3 (0.33)
Normal weight	394 (43.20)	64 (7.02)
Overweight	219 (24.01)	92 (10.09)
Obese	78 (8.55)	49 (5.37)
Smoking		
No	683 (75.64)	192 (21.26)
Yes	15 (1.66)	13 (1.44)
Physical activity		
Not active	25 (2.75)	31 (3.41)
Moderately active	385 (42.35)	136 (14.96)
Active	292 (32.12)	40 (4.40)
Alcohol consumption		
Not at all	314 (35.01)	89 (9.92)
≤ 1 per month	265 (29.54)	51 (5.69)
1–3 times per week	83 (9.25)	36 (4.01)
Every day	31 (3.46)	28 (3.12)

**Table 2:** Mean (SEM) values of the anthropometric indices categorised by gender and hypertension status

	Male			Female			
	Normal (n = 142)	Prehypertensive (n = 198)	Hypertensive (n = 96)	Normal (n = 174)	Prehypertensive (n = 192)	Hypertensive (n = 112)	p-value
SBP, n (%)	104.44 ( 6.51)	121.29 (7.94)	145.13 (16.36)	104.44 (6.51)	121.54 (7.48)	145.68 (15.95)	0.749
DBP, n (%)	68.41 (6.02)	77.79 (5.39)	96.05 (8.98)	68.41 (6.02)	78.37 (5.94)	93.42 (11.32)	0.8795
Weight, n (%)	66.30 (9.10)	72.28 (11.13)	77.97 (14.09)	64.72 (11.11)	69.45 (11.86)	72.98 (15.55)	0.0000
Height, n (%)	169.11 (7.69)	169.74 (7.25)	170.58 (7.48)	162.20 (7.34)	162.50 (7.07)	162.65 (7.14)	0.0000
WC, n (%)	83.48 (8.03)	87.99 (8.16)	93.64 (11.86)	86.32 (10.66)	90.39 (12.71)	93.40 (11.86)	0.0117
HC, n (%)	93.56 (9.12)	97.58 (8.70)	101.73 (12.73)	98.08 (12.76)	101.09 (12.89)	103.58 (13.23)	0.0000
WHR, n (%)	0.89 (0.067)	0.90 (0.04)	0.92 (0.04)	0.88 (0.07)	0.90 (0.06)	0.90 (0.05)	0.0028
WHtR, n (%)	0.49 (0.044)	0.55 (0.067)	0.22 (0.03)	0.21 (0.03)	0.22 (0 .03)	0.23 (0.03)	0.0000
PI, n (%)	13.75 (1.96)	14.85 (2.49)	15.69 (2.37)	15.17 (2.33)	16.28 (3.07)	16.94 (3.28)	0.0000
BAI, n (%)	24.57 (3.91)	26.24 (4.56)	27.68 (5.33)	29.54 (5.96)	30.96 (6.97)	31.99 (6.19)	0.0000
BMI, n (%)	23.18 ( 2.90)	25.12 (3.78)	26.72 (3.95)	24.55 (3.57)	26.34 (4.43)	27.51 (5.22)	0.0001
CI, n (%)	1.23 (0.11)	1.24 (0.09)	1.28 (0.13)	1.25 (0.10)	1.27 (0.13)	1.29 (0.10)	0.0007

SBP, systolic blood pressure; DBP, diastolic blood pressure; WC, waist circumference; HC, hip circumference; WHR, waist–hip ratio; WHtR, waist–height ratio; PI, ponderal index; BAI, body adiposity index; BMI, body mass index; CI, conicity index.

Table 3 presents the results of the correlation between the anthropometric indices with blood pressure. All anthropometric indices correlated significantly with systolic and diastolic blood pressure. BMI had the highest correlation coefficient, while CI had the lowest.

**Table 3:** Correlation between blood pressure, age and anthropometric variables

	SBP (r)	r^2^	p-value	DBP (r)	r^2^	p-value
BAI	0.18	0.03	0.0000	0.12	0.01	0.0004
BMI	0.33	0.11	0.0000	0.29	0.08	0.0000
WHtR	0.25	0.06	0.0000	0.21	0.04	0.0000
WHR	0.15	0.02	0.0000	0.19	0.04	0.0000
PI	0.27	0.07	0.0000	0.25	0.06	0.0000
WC	0.27	0.07	0.0000	0.22	0.05	0.0000
HC	0.24	0.06	0.0000	0.16	0.02	0.0000
CI	0.105	0.01	0.0014	0.08	0.01	0.0410

SBP, systolic blood pressure; DBP, diastolic blood pressure; BAI, body adiposityindex; BMI, body mass index; WHtR, waist–height ratio; WHR, waist–hip ratio; PI, ponderal index; WC, waist circumference; HC, hip circumference; CI, conicity index.

Table 4 lists the results of the predictive potentials of each individual anthropometric index in discriminating between hypertension and normotension, and between prehypertension and normotension. For hypertension, BMI, WHtR and WC had the strongest/highest predictive potential in both the male and female categories (WHtR was slightly higher). BMI and WHtR also performed relatively well in all age categories except for age category 2 (21–25 years) for BMI, and age categories 1 and 2 (≤ 20 years and 21–25 years) for WHtR. PI also showed a strong predictive power (AUC) in this regard but was lower than that of BMI (p < 0.05), WHtR and WC. There was no significant difference between the AUC of BMI and that of WHtR and WC. The predictive powers of the four indices were higher in male than female participants.

**Table 4:** Analysis of the predictive power of each index for hypertension and prehypertension

Anthropometric measures	Hypertension AUC	p-value	Prehypertension AUC	p-value
BAI^#^ Age category				
1	0.535	0.8185	0.542	0.5523
2	0.534	0.5274	0.525	0.4775
3	0.583	0.0229	0.500	0.9964
4	0.626	0.0034*	0.613	0.0642
BAI^#^ Gender				
Male	0.625	0.0002*	0.581	0.0009*
Female	0.594	0.0019*	0.564	0.0329*
BMI Age category				
1	0.727	0.0001*	0.563	0.3575
2	0.542	0.4923	0.571	0.0399*
3	0.686	0.0001*	0.622	0.0008*
4	0.589	0.0434*	0.674	0.0133*
BMI Gender				
Male	0.698	0.0001*	0.659	0.0001*
Female	0.622	0.0001*	0.609	0.0002*
WHtR Age category				
1	0.511	0.9172	0.559	0.3915
2	0.556	0.2618	0.515	0.6764
3	0.631	0.0002*	0.560	0.1080
4	0.601	0.0209*	0.615	0.1408
WHtR Gender				
Male	0.682	0.0001*	0.636	0.0001*
Female	0.624	0.0001*	0.572	0.0163*
WHR^#^ Age category				
1	0.623	0.3645	0.614	0.0839
2	0.502	0.9693	0.501	0.9817
3	0.643	0.0001*	0.619	0.0012*
4	0.525	0.5685	0.662	0.0876*
WHR^#^ Gender				
Male	0.645	0.0001*	0.562	0.0531*
Female	0.570	0.0208*	0.554	0.0753*
WC Age category				
1	0.508	0.9433	0.591	0.1634
2	0.550	0.3858	0.556	0.1038
3	0.607	0.0031*	0.551	0.1645
4	0.542	0.3421	0.580	0.3115
WC Gender				
Male	0.692	0.0001*	0.645	0.0001*
Female	0.616	0.0001*	0.584	0.0046*
PI^#^ Age category				
1	0.680	0.0008*	0.652	0.0223*
2	0.525	0.6617	0.547	0.1818
3	0.679	0.0001*	0.607	0.0043*
4	0.642	0.0008*	0.670	0.0132*
PI^#^ Gender				
Male	0.670	0.0001*	0.639	0.0001*
Female	0.619	0.0001*	0.599	0.0008*
HC^#^ Age category				
1	0.530	0.8371	0.649	0.0193*
2	0.552	0.3242	0.543	0.2095
3	0.565	0.0866	0.507	0.8576
4	0.535	0.4372	0.567	0.3003
HC^#^ Gender				
Male	0.646	0.0001*	0.602	0.0008*
Female	0.592	0.0036*	0.584	0.005*
CI^#^ Age category				
1	0.618	0.2350	0.556	0.3980
2	0.503	0.950	0.520	0.5602
3	0.527	0.4571	0.520	0.5915
4	0.539	0.3878	0.541	0.6106
CI^#^ Gender				
Male	0.592	0.0082*	0.541	0.2032
Female	0.558	0.0528	0.533	0.2773

*Statistically significant at p < 0.05; ^#^AUC significantly different from that of BMI. Age category 1 = ≤ 20 years, 2 = 21–25 years, 3 = 26–40 years, 4 = ≥ 41 years. BAI, body adiposity index; BMI, body mass index; WHtR, waist–height ratio; WHR, waist–hip ratio; WC, waist circumference; PI, ponderal index; HC, hip circumference; CI, conicity index.

For prehypertension, BMI, WC, PI and WHtR had higher predictive potentials for both genders, with BMI showing slightly higher power among the four indices. BMI and PI seemed to perform better in virtually all age groups than the other two indices.

Table 5 examined the possible linear relationship between the four best anthropometric indices and hypertension and prehypertension prevalence (risk). The anthropometric indices were considered as continuous variables to calculate prevalence ratios corresponding to one standard deviation change. The hypertension prevalence ratio increased by 15% (WHtR), 15% (WC), 14% (BMI) and 12% (PI) with one standard deviation increase in the corresponding anthropometric index on adjusting for gender, age, alcohol intake and physical activity; the prehypertension prevalence ratio increased by 4% (WHtR), 11% (WC),11% (BMI) and 6.7% (PI).

**Table 5:** Prevalence ratios corresponding to one standard deviation increase in anthropometric measures

Anthropometric index	Hypertension			Prehypertension		
	PR	95% CI	p-value	PR	95% CI	p-value
Model 1						
WHtR	1.44	1.3–1.56	0.000	1.15	1.08–1.22	0.000
WC	1.41	1.28–1.56	0.000	1.2	1.13–1.27	0.000
BMI	1.35	1.24–1.47	0.000	1.21	1.15–1.27	0.000
PI	1.32	1.21–1.44	0.000	1.17	1.11–1.23	0.000
Model 2						
WHtR	1.15	1.01–1.30	0.030	1.04	0.97–1.11	0.258
WC	1.15	1.03–1.29	0.017	1.11	1.03–1.19	0.002
BMI	1.14	1.01–1.27	0.028	1.11	1.04–1.10	0.001
PI	1.12	0.99–1.27	0.063	1.067	1.00–1.14	0.038

PR, prevalence ratio; WHtR, waist–height ratio; WC, waist circumference; BMI, body mass index; PI, ponderal index.

Model 1 was crude while model 2 was adjusted for factors such as age, smoking, alcohol consumption and physical activity.

Table 6 lists the cut-off points and Youden index J for the four best anthropometric indices in predicting hypertension and prehypertension. The best Youden index J was recorded in BMI and WHtR for all categories but this was not strikingly distinct.

**Table 6:** Cut-off points for anthropometric indices in predicting hypertension and prehypertension

	Hypertension		Prehypertension	
	Male	Female	Male	Female
BMI				
Cut-off point	24.49	24.44	23.62	28.01
Sensitivity	72.92	74.11	64.65	31.05
Specificity	60	48.9	64.79	58.51
Youden index J	0.33	0.20	0.29	0.20
WHtR
Cut-off point	0.55	0.508	0.50	0.60
Sensitivity	48.96	81.25	58.59	25.79
Specificity	83	40.38	61.97	93.68
Youden index J	0.33	0.22	0.21	0.2
WC				
Cut-off point	91.44	96.52	82.55	96.52
Sensitivity	53.13	40.18	71.21	32.11
Specificity	81.47	76.65	50.7	86.21
Youden index J	0.35	0.17	0.22	0.18
PI
Cut-off point	14.45	16.38	13.69	17.65
Sensitivity	70.83	57.65	71.21	28.42
Specificity	57.65	67.3	57.75	87.36
Youden index J	0.28	0.24	0.30	0.16

BMI, body mass index; WHtR, waist–height ratio; WC, waist circumference; PI, ponderal index.

The effect of other anthropometric indices on BMI prevalence ratio, as well as its hypertension predictive power is shown in Table 7. On average, each unit increase in BMI was associated with a 26 and 14% increase in prevalence ratio for hypertension in the male and female categories, respectively (model 1 p < 0.05 in most cases). There was about a 17 and 4% increase in prevalence ratio associated with one unit increase in BMI in model 2 in males (p < 0.05) and females (p > 0.05), respectively.

**Table 7:** Gender-specific prevalence ratios and AU Cs of BMI for hypertension

Male	PR	95% CI	p-value	% PR change	AUC	% AUC change	p-value
Model 1							
BMI	1.10	1.07–1.14	0.000	–	0.6978	–	
BMI + WHtR	1.03	1.0–1.10	0.206	6.56	0.7103	1.78	0.0011
BMI + WC	1.02	0.97–1.08	0.341	7.55	0.7104	1.79	0.0000
BMI + PI	1.26	1.09–1.45	0.002	13.58	0.6963	0.22	0.0258
Model 2							
BMI	1.06	1.02–1.10	0.002		0.8301	0.00	
BMI + WHtR	1.04	0.99–1.09	0.142	1.90	0.8319	0.22	0.1850
BMI + WC	1.03	0.98–1.08	0.302	2.87	0.8373	0.86	0.0206
BMI + PI	1.17	1.01–1.36	0.037	9.87	0.8399	1.17	0.0381
Female				
Model 1
BMI	1.07	1.04–1.10	0.000		0.6221		
BMI + WHtR	1.05	1.01–1.10	0.023	1.89	0.6238	0.27	0.4532
BMI + WC	1.06	1.0–1.09	0.034	0.94	0.6245	0.39	0.2316
BMI + PI	1.14	1.01–1.29	0.027	6.34	0.6202	0.31	0.2527
Model 2							
BMI	1.01	0.98–1.05	0.400		0.7468		
BMI + WHtR	1.02	0.98–1.07	0.359	0.99	0.7477	0.12	0.6346
BMI + WC	1.02	0.97–1.06	0.450	0.99	0.7465	0.04	0.8587
BMI + PI	1.04	0.94–1.16	0.437	2.93	0.7474	0.08	0.5754

PR, prevalence ratio; BMI, body mass index; WHtR, waist–height ratio; WC, waist circumference; PI, ponderal index. Model 1 was crude while model 2 was adjusted for factors such as age, gender, smoking, alcohol consumption and physical activity. % change in PR = 100 × absolute [ln (PR_BMI_/PR_test variables_)]; test variables were BMI + WC, BMI + WHtR, or BMI + PI.^28^ % change in AUC = 100 × absolute [ln (AUC_BMI_/AUC_test variables_)]; test variables were BMI + WC, BMI + WHtR, or BMI + PI.^28^

The combination of WC, WHtR and PI did not change the prevalence ratio beyond 10%, except for PI, which gave percentage changes of 13.58 and 9.87% for model 1 and 2, respectively in males. The changes due to the addition of WC and WHtR were generally decremental, while that of PI was incremental. On average, the changes in PR were higher in model 1 compared to model 2. There was an increase in model fit (AUC) when WC, WHtR or PI were used in model 1 and 2, except for PI in model 1, which resulted in a slight decrease in AUC when compared with BMI only. However, none of the percentage changes in the AUCs of each model was < 2% (p < 0.05 in almost all models for males and > 0.05 in all models for females.)

## Discussions

To our knowledge, this study is the first to comprehensively compare the performance of a large set of anthropometric indices as correlates and potential predictors of risk for hypertension and prehypertension in a typical Nigerian (West African) population. We analysed the performance of some anthropometric indices of obesity as potential predictors of hypertension and prehypertension.

The mean values of the following anthropometric measures, BMI, WC, HC, CI and BAI were significantly higher in women. This could have been attributed to the general inactivity of women in this population. The mean values of all the anthropometric indices studied were higher in the prehypertensive and highest in the hypertensive participants relative to the normotensive participants. This is an indication that participants with a higher obesity index tend to have high blood pressure values. This finding is consistent with reports from previous studies.^5^,^8^,^26^

The correlation analysis showed that all the studied anthroprometric indices were correlated with SBP and DBP. BMI, WC, PI and WHtR had correlation coefficients greater than 0.25, while BAI and CI correlated poorly with blood pressure. Our results also showed BMI, WC, WHtR and PI performed best as potential predictors of the risk for hypertension on comparing respective AUCs from ROC curve analysis. The prevalence ratios for general obesity index were lower than that of central obesity in both the crude and adjusted models, however these differences were not large enough to suggest that central obesityindex (WC or WhtR) outperformed general obesity index (BMI) in this study. There was no significant difference between the performances of BMI, WC and WHtR in predicting risk for hypertension. A similar finding was reported previously by Lee and co-workers.^10^

BAI and HC showed a fair performance in predicting hypertension and prehypertension risk. CI had a poor predictive power for hypertension and totally lacked the capacity to distinguish prehypertensive cases from normotensive cases. The results of the ROC and correlation analyses were consistent and showed similar trends.

Anthropometric indices (BMI, WC, WHtR and PI), which had higher correlation coefficients with blood pressure (SBP and DBP), had very high AUCs that were statistically significant (p < 0.05). The reverse was true for poorly correlated anthropometric indices such as BAI, WHR and CI. CI was the poorest correlate of hypertension and prehypertension (AUC = 0.5, p > 0.05). BMI, WHtR and WC emerged the best predictors of hypertension and prehypertension in this study.

These findings conform with and confirm the findings of Silva et al.^27^ in Brazillian women and men, Sanchez-Viveros et al.^28^ in Mexican women and men, and Uhernik et al.^29^ in Croatian men and women. They differ from those of Feldstein et al.^18^ in Argentina and Li et al.^19^ in Australia where none of BMI, WC or WHtR emerged as the best predictors of hypertension or prehypertension. These results also provide evidence to support the findings that suggested the superiority of WC and BMI over BAI.^30^

As mentioned above, epidemiological studies on the predictive potentials of anthropometric indices for hypertension and cardiovascular-related diseases are limited in Nigeria. Okafor et al.^23^ reported WC was a better predictor of obesity and hypertension than WHR in a population with similar characteristics to our study population, while Sonuyi and co-workers^31^ reported normative values of selected anthropometric variables in Lagos, Nigeria. Both findings were consistent with our results.

The differences in the results of some of the previous studies mentioned could have been attributed to differences in the characteristics of the populations. Evidence of racial/ethnic, gender and age variations in anthropometry is well established.^32^ Sakurai et al.^3^ reported that the percentage body fat in Asians, as measured by dual-energy X-ray absorptiometry is greater than in African Americans and whites with a similar BMI. Variations in the level of leptin (the product of the gene largely responsible for obesity) across different ethnic groups and races is also well established.^33^ Human body composition is evidently a result of complex multifactorial interactions between lifestyle, culture, environmental and genetic differences,^33^ which vary from place to place and impact differently on the results of studies in different populations.

Secondly, rigours, technicalities and lack of universally accepted standards in measuring some anthropometric measures could account for some of the reported differences in different studies.^34^ Our study also provided evidence to suggest that the predictive potential of anthropometric indices may vary with age. BMI, PI and WHtR performed well in predicting risk for hypertension and prehypertension in three age categories (≤ 20, 26–40 and ≥ 40 years), while BAI was better in one age category (≥ 40 years). HC and CI were not particularly outstanding in any of the age categories. This differential performance in different age categories could also account for the variations in the results from different studies.

Our predicted cut-off points for some of the anthropometric predictors of hypertension were somewhat similar to that proposed by the WHO and other studies^8^,^10^,^11^,^35^ in Korean, Brazilian and Pakistani populations, respectively. However, the cut-off points for WC and WHtR were higher in our study when compared to the WHO cut-off value. This could be attributed to the higher WC and lower height of females in the population.Africans and Westerners have quite distinct anthropometry occasioned by differences in culture, environment, genetics, nutrition as well as economy. Most of the recommended cut-off points are more representative of Western populations. The cut-off points for the anthropometric indices in our study differed markedly in women for prehypertension; the predicted cut-off points were higher in women and lower in men.

The performance of the anthropometric indices in predicting both conditions differed by gender in this study. All the indices studied tended to predict risk for hypertension and prehypertension better in males than in females. These differences have also been corroborated by previous independent studies.^3^,^7^,^36^ There is evidence that fat distribution in men and women differs. Visceral fat is more dominant in men and subcutaneous fat in women. This may provide an explanation for the existence of gender differences in the performance of anthropometric indices. Visceral fat has a stronger association with metabolic abnormalities than subcutaneous fat,^3^ and this could also explain why we found a higher risk for hypertension with regard to obesity in males than females in this study.

Our study presents evidence that the relationship between obesity and the two conditions, hypertension and prehypertension, differed in terms of the performance of anthropometric indices. This is to be expected as prehypertension has been described by JNC-7 as a new category of hypertension with high risk for the development of clinical hypertension.^37^ Most physicians describe it as a pre-sign to clinical hypertension. However, it has also been established that it presents a high risk for cardiovascular and coronary heart disease, even without the development of clinical hypertension and should be given adequate interventional attention by promoting early lifestyle modification to prevent progression to blood pressure or other related chronic diseases.

Prehypertension, unlike hypertension, is most often without symptoms, but both conditions have been shown to share similar risk factors, such as age, overweight, obesity and hyperlipidaemia, but to different extents. The odds of the risk factors are usually higher in hypertension than in prehypertension. The association of higher cardiac and haemodynamic characteristics with hypertension and prehypertension has also shown similar trends.^38^ These minor differences may explain the differences in predictive potentials of some anthropometric indices for hypertension and prehypertension.

Lee and Kim in their studies suggested the use of combined anthropometric indices in models to improve the predictive potential for hypertension or related diseases. Our study shows WC and WHtR added to the prediction of hypertension using BMI when included in a model, however, this contribution was not statistically significant using our decision rule. The association of WHtR, WC and BMI with hypertension prevalence rate was statistically significant and similar in magnitude on adjusting for age, gender, physical activity and alcohol intake, but differed for prehypertension. WC and BMI had a stronger (significant p < 0.05) association with prehypertension relative to PI and WHtR (p > 0.05). This was consistent with the ROC curve analysis result. This reinforces the fact that BMI, WHtR and WC were equally good indicators of hypertension in this population; none significantly outperformed the other.

Overall, the mechanism of association of general obesity (BMI and PI) with hypertension may differ from that of central obesity (WHtR and WC) with hypertension. As mentioned, the changes due to the addition of WC and WHtR were generally decremental, while that of PI was incremental when WC, WHtR or PI was included in a model with BMI. This is to be expected as WC and WHtR were strongly correlated (τ = 0.95; p < 0.0001) and BMI and PI were also correlated (τ = 0.98; p < 0.0001). The correlation between BMI, WC and WHtR (τ = 0.60, τ = 0.64 respectively) was equally strong. The normalisation of BMI by a factor of 1/height (m) to give PI did not improve the predictive power of BMI, as traditional BMI outperformed PI in this study.

## Limitations

The limitations of the present study include: firstly, the cross sectional nature of the study precludes conclusion about a cause–effect relationship. A longitudinal study in this regard is required; secondly, our study population was drawn from a particular religious group in a state in south-eastern Nigeria. This may not be a true representation of the Anambra state population and therefore limits the application of our findings to other populations. However, it is noteworthy that the population not captured represented a negligible proportion of the major population. Thirdly, our overall sample size was large but on categorisation by age group, some age groups had small sample sizes, which may have limited our statistical power to detect better performance in some age categories.

Despite these limitations, the study has some strengths. The study population was large and typical of an African population, where there has been dearth of data of this sort. The anthropometric and blood pressure measures were standardised.

## Conclusion

This study showed BMI, WC, WHtR and PI were strongly associated with blood pressure and were better potential predictors of risk for hypertension and prehypertension than the other indices tested. They performed well independently and there was no evidence to show that WC, WHtR or PI outperformed or statistically added to the prediction power of BMI. Their prediction potentials were better in the male gender and in predicting risk for hypertension than for prehypertension.

In practice, these anthropometric measures are surrogate measures of body fat and are cost free, practical and easy to interpret for healthcare providers and lay people.^39^ In the context of developing countries, indices of obesity (both general andabdominal) could be used simultaneously but independently to predict risk for both conditions, since they both performed well and possibly define different mechanisms of the association of obesity with hypertension and other cardiovascular disorders.
